# Analysis of wearable time series data in endocrine and metabolic research

**DOI:** 10.1016/j.coemr.2022.100380

**Published:** 2022-08

**Authors:** Azure D. Grant, Thomas J. Upton, John R. Terry, Benjamin L. Smarr, Eder Zavala

**Affiliations:** 1 Helen Wills Neuroscience Institute, University of California, Berkeley, 94720, United States of America; 2 Laboratories for Integrative Neuroscience and Endocrinology, University of Bristol, Bristol, BS1 3NY, United Kingdom; 3 Centre for Systems Modelling & Quantitative Biomedicine, University of Birmingham, Edgbaston, B15 2TT, United Kingdom; 4 Department of Bioengineering, University of California, San Diego, 92093, United States of America; 5 Halıcıoğlu Data Science Institute, University of California, San Diego, 92093, United States of America

**Keywords:** Hormone dynamics, Wearables, Time series analysis, Computer algorithms, Personalised medicine, Precision medicine

## Abstract

Many hormones in the body oscillate with different frequencies and amplitudes, creating a dynamic environment that is essential to maintain health. In humans, disruptions to these rhythms are strongly associated with increased morbidity and mortality. While mathematical models can help us understand rhythm misalignment, translating this insight into personalised healthcare technologies requires solving additional challenges. Here, we discuss how combining minimally invasive, high-frequency biosampling technologies with wearable devices can assist the development of hormonal surrogates. We review bespoke algorithms that can help analyse multidimensional, noisy, time series data and identify wearable signals that could constitute clinical proxies of endocrine rhythms. These techniques can support the development of computational biomarkers to support the diagnosis and management of endocrine and metabolic conditions.

## Highlights

• Endocrine and metabolic rhythms often become misaligned during disease.
• Quantifying endocrine rhythm variability is key to personalise health interventions.
• Multimodal, high-frequency sampling can help disentangle endocrine systems function.
• Multi-dimensional wearable datasets can constitute surrogates of endocrine rhythms.
• Digital phenotyping is now widely accessible through rhythmic analysis algorithms.

## Introduction

The coordination of hormonal rhythms plays a key role in sustaining health. Combining experimental physiology with mathematical and computational techniques contributes to our understanding of the mechanisms underpinning rhythmic hormonal secretion, how rhythms are decoded by target tissues, and their responses to perturbations [1]. This allows us to understand how regulatory mechanisms ensure that hormone fluctuations achieve dynamic equilibration, and how disruptions of these mechanisms lead to rhythm misalignment and disease [2,3]. Phenomenologically, these techniques allow more precise descriptions of the endocrine systems over time, and are foundational to the development of digital health tools, as in diagnostic and screening algorithms [4].

Although mathematical models can provide mechanistic insight [1,5], translating this understanding into clinical solutions remains a significant challenge. A key step for advancing personalised medicine is to characterise the intra-individual and inter-individual variability over relevant time scales [4]. It is important to accurately match a person's hormonal rhythms to their chronotype (diurnal propensity toward sleep and certain behaviours, as in early birds vs. night owls) [6] and glucotype (distinct patterns of glycemic responses over time, as in “gluconormal mean but moderate variability”) [7], and to assess the impact of disruptions such as chronic stress, shift work, and social jet lag [8] on successful clinical interventions. The scarcity of high-frequency data about hormone fluctuations in physio-pathological scenarios makes it difficult to properly quantify variability. This is mainly because high-frequency measurement of analytes is expensive, time consuming, and burdensome for patients and researchers. For example, simultaneous high frequency measurement of analytes such as cortisol, melatonin and glucose has been mainly limited to serum samplings [9,10]. Additionally, collected samples may require specific analysis methods that are not widely available, or non-ambulatory biosampling technologies, limiting the scope of research studies to lab or hospital settings.

**Figure 1:**
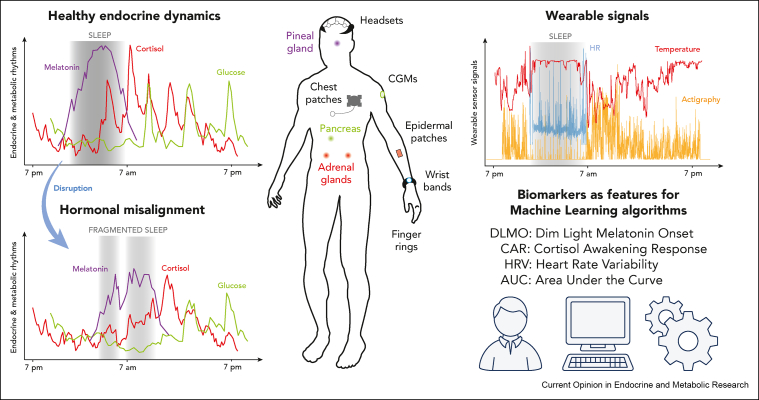
Endocrine and metabolic rhythms have been widely observed, and are key to sustain healthy states. Left: Misalignment of rhythms in signalling molecules (e.g., melatonin, cortisol, and glucose) is strongly associated to physio-pathological changes such as dietary changes, pregnancy, shift work, social jet lag, systemic inflammation and chronic disease. Top right: Wearable device signals (e.g., heart rate (HR), body temperature, and accelerometer-derived actigraphy) may act as continuous surrogates of endocrine rhythms specific to an individual's internal time (e.g., chronotype, glucotype). Bottom right: Endocrine and wearable signals are commonly described using metrics such as dim light melatonin onset (DLMO), cortisol awakening response (CAR), heart rate variability (HRV) and area under the curve (AUC). Mathematical and computational techniques, including machine learning, can help identify these metrics as clinical proxies (e.g., computational biomarkers) to support early diagnosis and management of disease.

Minimally invasive ambulatory biosampling is making high-frequency measurements more accessible, including subcutaneous microdialysis [11] (Box 1), continuous glucose monitoring systems (CGMs) [12], and epidermal sensors [13,14]. Additionally, small, cheap, non-invasive wearable technologies now integrate highly-sensitive, low-latency sensors, with improved memory and battery, attracting the interest of the biomedical research community [15,16]. The hope is that the multi-dimensional streams of data provided by these devices can translate into clinically actionable insights about a patient's physiological dynamics (Figure 1). With these developments comes the need to map features of hormonal dynamics onto signals from non-invasive sensors, as well as quantifying the variability in such relationships. The adoption of novel technologies within endocrine and metabolic research will need to get past “black boxes” to ensure a broad usability across populations. Wearables are increasingly used across biomedical applications [4,17,18], but the physiology underlying the wearable data often remains cryptic. Here, we focus on the challenges associated with identifying correlates of hormonal dynamics through minimally invasive and non-invasive wearable technologies.

Box 1 **Example 1: Subcutaneous ambulatory biosampling.**

Widespread adoption of continuous glucose monitoring systems, which reveal dynamic changes in glucose, has improved the treatment of type 1 diabetes. Continuous glucose monitors have helped improve glycemia [31] and reduce psychosocial stress [32] when used in concert with insulin pumps to form artificial pancreas systems [33]. Other endocrine systems are beginning to benefit from similar approaches. Cortisol, which is secreted with both circadian and ultradian rhythmicity [2], is typically measured at single time points, making clinical interpretation difficult. Further, in conditions of chronic cortisol deficiency (e.g., autoimmune adrenal insufficiency) standard replacement is at best a crude representation of normal physiology. As a result, patients suffer poorer quality of life and remain at increased risk of health complications and premature death [34]. The development of at-home wearable technology for high-frequency measurement of cortisol [11] promises to revolutionise the management of glucocorticoid disease. The ULTRADIAN trial (in progress, NCT02934399) aims to prove the technology by describing the normal variation of cortisol and many other adrenal steroids in comparison with endocrine diseases in >300 healthy volunteers and patients. Data from 24-h profiles will be interpreted using mathematical approaches that integrate multiple features in the time series (i.e., classical statistical approaches, as in mixed effects models, or more modern approaches, as in machine learning), correlated with events such as sleep and meals times, providing novel insights and an offer of a more personalised diagnosis and treatment plan.

Alt-text: Box 1

## Identifying clinical proxies of hormonal dynamics using wearables

Studies in healthy populations and patient cohorts have established correlations between hormonal dynamics and physiological variables measured through wearables (Table 1). Using algorithmic processing, combinations of certain wearable signals may reflect clinically-relevant dynamic features of hormonal regulation. However, such algorithms often fail to generalize across heterogeneous populations, limiting real-world applicability outside of specific cohorts and study conditions. Additionally, hormonal dynamics are complex, containing multiple rhythmic frequencies (e.g., circadian and ultradian), rapid responses to perturbations followed by dynamic equilibration (homeostasis), and slow adaptations to physio-pathological states (allostasis), all of which also differ across individuals. The wide adoption of wearables across diverse populations provides an opportunity to investigate the variability in endocrine dynamics, which will hopefully enable development of precision algorithms for specific cohorts or conditions.

**Table 1:** Selected recent studies linking wearable device outputs with **a)** behavioural parameters, and **b)** with simultaneous measurement of hormonal and metabolic analytes.

Type	Reference	Measured endocrine parameters and wearable signals
a) on behavior	[19]	Activity, heart rate, and sleep timing.
	[20]	Peripheral skin temperature and circadian sleep/wake cycles.
	[21]	Actigraphy for sleep-wake classification.
	[22]	Temperature changes at sleep onset across menstrual and circadian phases.
b) on hormones	[23]	Heart rate variability (HRV) biomarkers to estimate circadian melatonin levels and body temperature.
	[24]	Ultradian HRV and temperature anticipate the luteinizing hormone surge.
	[25]	Combining chronotype questionnaires, body temperature, dim light melatonin onset (DLMO) and actigraphy to estimate circadian phase.
	[26]	HRV and salivary cortisol for identifying the stress response based on adverse childhood experience.
	[27]	Detection of hypoglycemic events from electrocardiograms (ECGs).
	[28]	Multisensor device integrating actigraphy, galvanic skin response, skin temperature, ECG paired with CGMs to estimate glucose levels.

Finding associations between hormonal and digital phenotypes is particularly challenging because endocrine systems are entangled (e.g., temperature rhythms reflect the combined output of several endocrine axes). In fact, recent mathematical and computational approaches account for how the coupling mechanisms among endocrine systems can influence their dynamics [29]. Thus, added modalities (i.e., multiple recorded channels/dimensions) and high-frequency sampling (i.e., continuity) provide an information advantage. Multimodality and continuity has already revealed useful patterns (e.g., chronotypes, glucotypes, pregnancy detection), and more modalities are expected to enhance this further, leading to increased precision based on *physiotypes*. Finally, multimodality also allows for error correction based on comparisons across sensors [30], and for inferring oscillation phase. This is especially relevant as we map high-frequency signals, where uncertainty is high.

## Computational techniques to improve interpretation of endocrine and wearable data

The methods chosen to assess rhythmicity in wearable and endocrine data can greatly influence the conclusions of a study. This choice can mean the difference between detecting or failing to detect rhythmicity, and can impact assessment of frequency composition and coupling among signals. Here, we provide a concise overview of common methods for biological signal analysis, their benefits and drawbacks, examples of appropriate use, and published tools that aid in application (for an introduction, see the study by Forger et al. [35]). Below, we outline: 1) pulse detection algorithms, 2) wavelets and wavelet coherence, and 3) ensemble empirical mode decomposition (EEMD).

### Pulse detection algorithms

These algorithms (series of predetermined steps and equations) take a time series as an input, and output locations of pulses and inter-pulse-intervals (IPIs). While a “pulse” can be defined simply as a local-maximum, it is often difficult to algorithmically distinguish peaks that are biologically-driven from peaks arising from noise, or oscillations not driven by the oscillator of interest. Criteria for pulse detection include pulse amplitude and frequency, but baselines may themselves change based on context, as in underlying inflammation or phase of a longer oscillation (e.g., ovulatory cycles). Historically applied pulse detector algorithms in neuroendocrinology include Cluster, Santen and Bardin, Regional Dual Threshold, Pulsar, Cycle Detector, Ultra, and AutoDecon [36].

AutoDecon is an example of a fully automated algorithm based on mathematical models of real endocrine events; given a time series, it solves for the number of hormonal pulses apparent given an optimal fit of the model (for full details, see the study by Vesco et al. [32]), as well as initial parameter estimates. It does this while simultaneously performing deconvolution [36]. The deconvolution uses a mathematical model accounting for hormonal secretion and elimination processes, fits it to experimentally observed time series data, and performs rigorous statistical tests to automatically find the optimal number of secretion events. AutoDecon includes a triage module to automatically remove secretion events deemed to be statistically non-significant. Other pulse detection algorithms (e.g., Cluster) work by similar statistical principles: finding a statistically significant increase in a group of values, followed by a statistically significant decrease in the subsequent cluster. Improvements to pulse detection algorithms continue to be published, incorporating criteria for pulse shape, a-priori period estimation (if available), and the combination of global and context-dependent criteria for determining sufficient pulse amplitude [37].

#### Advantages and drawbacks

Advantages of these programs include ease of use; and a degree of standardization among researchers when parameters, sampling frequency, and sampling duration are reported. However, different algorithms yield different curves of pulse frequency estimation vs. sampling interval, likely due to different methods of modelling variance and different false positive and false negative rates [36].

### The continuous/analytic wavelet transform (CWT/AWT)

Wavelet analysis is widely used in chronobiology, and provides advantages over older techniques (e.g., Pulse detection algorithms, Fourier transformations) [38,39] for analysis of signals with time-varying rhythmic composition and/or non-sinusoidal structure. Below, we focus on continuous wavelet transforms (CWTs) using the Morse wavelet, commonly applied to analysis of circadian and ultradian rhythms. For toolboxes on getting started with wavelet analysis see the studies by Leise et al., Lilly et al., Lee et al. [39∗, 40, 41], and the MATLAB Wavelet Toolbox (mathworks.com/products/wavelet).

A wavelet is a waveform with amplitude diminishing to 0 in both directions from centre, thereby possessing frequency, central amplitude, and position. By comparison, classic frequency analysis done with Fourier Transforms rely on infinite sine waves, which reduces flexibility in terms of precise shape, and lack a centre, making them ideal for confidence in the composition of stationary signals. Fast-Fourier Transforms trade precision for limited localization, but cannot achieve the same level of localization as CWTs, and therefore cannot as precisely locate changes in non-stationary signals (i.e., signals where frequency changes with time). CWTs correlate a time series of interest with scaled wavelet functions centred at each subsequent moment in time, detecting time-varying power-spectrum density profiles. At each moment, periodicity of the signal of interest is estimated by finding the scale(s) that maximize the correlation between the time series and a scaled wavelet. The absolute value of this “wavelet ridge” is an instantaneous frequency and amplitude estimation of the signal at each point [38,42]. Moreover, wavelets can take on an infinite number of forms based on the waves superimposed to create the wavelet (e.g., Mexican hat, square wave, Morse). This enables the experimenter to match the structure of the time series of interest to a similar wavelet [43,44]. Wavelet outputs can be compared to identify “wavelet coherence,” which reflects synchrony and phase-relationship of two analyzed signals [45,46].

#### Advantages and drawbacks

Wavelet analysis is useful for time series that are variable in time frequency composition, include many repetitions of the rhythms of interest, and are relatively free of data gaps. By averaging/linearizing bands of the wavelet matrix representing frequencies of interest, one can create a time series of “rhythmic power” which itself can be analysed as a signal, or at discrete points in time relative to events of interest. Wavelet analysis is not without drawbacks. Generating a wavelet matrix, especially on long, high-frequency time series, is computationally intensive. Wavelets also exhibit edge effects, meaning that data within an oscillatory period of a data gap, at the beginning or end of a time series, or at artificial data junctions, is corrupted.

### Ensemble empirical mode decomposition

EEMD is an unbiased method of separating meaningful oscillations from noise that can subsequently be combined with other methods of frequency analysis. This method results in the extraction of intrinsic mode functions (IMFs) that compose the parent signal. EEMD is not dependent on any specific underlying waveform, and the resultant IMFs may vary in rhythmic features like amplitude or frequency [47,48]. Briefly, local extrema are identified, and local maxima and minima are interpolated by cubic splines to create an upper and lower envelope. The mean of the upper and lower envelopes is then subtracted from the initial data, creating a first IMF. These steps are repeated recursively, with each derived IMF acting as the initial data for the generation of the next. To overcome issues arising from cases in which timescales within a signal are mutually influential (as occurs when phase, amplitude, and waveform of ultradian rhythms are impacted by time of day), an additional practice is added. The “ensemble” in EEMD refers to adding noise to an average set of IMFs across repetitions of the process described above (see noise-assisted data analysis [47]). White noise is added separately to each iteration, and as the mean of each “ensemble” is treated as the true result, the signal itself persists while the effects of noise are cancelled out. Hilbert spectral analysis [49] (or other signal processing methods) can then be used to calculate the instantaneous frequency of each of the IMFs over time, resulting in a periodicity–time plot of signal amplitude over time (the Hilbert spectrum).

#### Advantages and drawbacks

The advantage of EEMD is in its objective decomposition and denoising of the signal of interest. Rather than filtering out or ignoring frequencies outside predetermined bands of interest, the IMFs generated by this process contain an unbiased representation of the composition of the signal. With EEMD specifically, mutually influential frequencies within a signal can be separated [47,50]. As with wavelet analysis, IMFs are subject to edge effects.

## Future directions

As continuous data becomes easier to attain through advances in sensor technology and the growth of the wearable market, broader adoption of signal processing techniques will ensure more efficient information extraction from wearable datasets. Continuous data composed of oscillating signals are not amenable to linear statistical comparisons (e.g., mean, standard deviation), which rest on assumptions that variance is random, not dependent or structured in time. Detecting endocrine changes through proxies extracted from wearables can inform research on human populations. Such research can be informed by related work using wearables to improve precision in the detection of illness involving changes to endocrine dynamics (Box 2) [57]. Systematic comparison of different techniques (those described here, and others as they emerge) could provide a reference to help researchers select appropriate analyses for specific data sets.

Box 2 **Example 2: Interfacing neuroendocrinology and neurology.**

Many people with epilepsy report their seizures being triggered by stressful stimuli and menstruation [51], and this appears linked to fluctuations in related hormonal systems. For example, fluctuations in the level of salivary cortisol are associated with the rates of epileptiform discharges in several human participants [52]. This observation builds on work that has shown that cortisol rapidly and reversibly alters the excitability of neurons [53] and that high doses of corticosterone increase the number of spike-wave discharges in a rodent model of absence seizures [54]. Further, seizures are the main cause of death in pregnant women with epilepsy [55]. Alterations in the cycling levels of oestrogen and progesterone can disrupt the balance of neuronal excitability and this is associated with increased occurrence of seizures [56]. As wearable technologies mature, algorithms which reveal proxies of fluctuating hormone levels from non-invasive measurements offer significant potential to advance research into a causal role for hormonal fluctuations as generative mechanisms of seizures. Understanding these dynamic mechanisms opens the door for novel diagnostic and treatment options not currently possible.

Alt-text: Box 2

## Declaration of competing interest

ADG, TJU, BLS and EZ declare that they have no known competing financial interests or personal relationships that could have appeared to influence the work reported in this article. JRT is co-founder of Neuronostics Ltd.
